# The role of Huidouba in regulating skeletal muscle metabolic disorders in prediabetic mice through AMPK/PGC-1α/PPARα pathway

**DOI:** 10.1186/s13098-023-01097-8

**Published:** 2023-07-01

**Authors:** Yu Tian, Dongxu Shi, Haiying Liao, Binan Lu, Zongran Pang

**Affiliations:** 1 School of Pharmacy, Minzu University of China, Beijing, PR China; 2 Key Laboratory of Ethnomedicine, Minzu University of China), Ministry of Education, Beijing, PR China

**Keywords:** Prediabetes, Skeletal muscle, Huidouba, AMPK

## Abstract

Prediabetes is a transitional state between normal blood glucose levels and diabetes, but it is also a reversible process. At the same time, as one of the most important tissues in the human body, the metabolic disorder of skeletal muscle is closely related to prediabetes. Huidouba (HDB) is a clinically proven traditional Chinese medicine with significant effects in regulating disorders of glucose and lipid metabolism. Our study aimed to investigate the efficacy and mechanism of HDB in prediabetic model mice from the perspective of skeletal muscle. C57BL/6J mice (6 weeks old) were fed a high-fat diet (HFD) for 12 weeks to replicate the prediabetic model. Three concentrations of HDB were treated with metformin as a positive control. After administration, fasting blood glucose was measured as an indicator of glucose metabolism, as well as lipid metabolism indicators such as total triglyceride (TG), low-density lipoprotein (LDL-C), high-density lipoprotein (HDL-C), free fatty acid (FFA), and lactate dehydrogenase (LDH). Muscle fat accumulation and glycogen accumulation were observed. The protein expression levels of p-AMPK, AMPK, PGC-1α, PPAR-α, and GLUT-4 were detected. After HDB treatment, fasting blood glucose was significantly improved, and TG, LDL-C, FFA, and LDH in serum and lipid accumulation in muscle tissue were significantly reduced. In addition, HDB significantly upregulated the expression levels of p-AMPK/AMPK, PGC-1α, PPAR-α, and GLUT-4 in muscle tissue. In conclusion, HDB can alleviate the symptoms of prediabetic model mice by promoting the AMPK/PGC-1α/PPARα pathway and upregulating the expression of GLUT-4 protein.

**Supplementary Information:**

The online version contains supplementary material available at 10.1186/s13098-023-01097-8.

## Introduction

With an aging population and the prevalence of unhealthy lifestyles, diabetes has become one of the fastest growing diseases of the 21st century. According to the International Diabetes Federation (IDF), the number of adults with diabetes worldwide had been expected to reach 537 million in 2021, and by 2045, 783 million will be affected [1]. The global prevalence of diabetes has increased alarmingly, among which type 2 diabetes (T2DM) is the main type. At the same time, there is a huge reserve army of people with T2DM, namely prediabetes. According to a survey, 70% of people with prediabetes will eventually develop T2DM [2]. Prediabetes is characterized by impaired glucose regulation (IGR), classified as impaired fasting glucose (IFG) and impaired glucose tolerance (IGT) (fasting blood glucose (FBG) ≥ 6.1 mmol/L but < 7.0 mmol/L; 2 h post-glucose blood glucose < 7.8 mmol/L is IFG; 2 h post-sugar blood glucose ≥ 7.8 mmol/L, but < 11.1 mmol/L is IGT; or a combination of both.) [3]. Prediabetes is a transitional state between normal blood glucose levels and diabetes, but it is also a reversible process. Timely intervention in prediabetic people will prevent or slow down the occurrence and development of T2DM. Currently, the pathogenesis of prediabetes is not yet clear, but most cases are believed to be related to insulin resistance (IR) and abnormal glucose and lipid metabolism. In the state of a high islet level, IR and glucose regulation are impaired, causing the disturbance of glucose and lipid metabolism and a rapid increase in the glucose level, resulting in elevated blood sugar [4]. Many proteins play an important role in the development of prediabetes to T2DM, such as adenosine 5’-monophosphate-activated protein kinase (AMPK), glucose transporter-4 (GLUT-4), and peroxisome proliferator-activated receptor (PPAR) [5, 6]. AMPK is known as a regulator of cellular energy. Under normal physiological conditions, when the intracellular adenosine-monophosphate/adenosine-triphosphate (AMP/ATP) ratio increases, AMPK is phosphorylated and activated [7]. Studies have shown that in the T2DM state, AMPK is considered to be an important protein kinase that regulates lipid metabolism disorders in blood and glucose transport and utilization in skeletal muscle by exercise or muscle contraction [8–10]. PPARα is another important protein which has unique effects on lipid metabolism and glycemic control [11]. It can control genes involved in fatty acid oxidation (regulating blood lipid levels), improve dyslipidemia, regulate glucose homeostasis, and affect energy metabolism balance [12–14]. PPARα can be activated by AMPK signaling to upregulate expression, but induction of PPARα gene targets requires the interaction of PPARα and PGC-1α as well as complexes with other enzymes and coactivators. The formation of such complexes appears to be necessary for complete transcriptional induction of PPARα gene targets [15]. One study found that in addition to PPARs, AMPK signaling also enhanced PGC-1α expression, induced glucose-consuming mitochondrial respiration, and increased glucose uptake in muscle cells (GLUT-4 upregulation), lowering blood glucose levels [16]. Moreover, studies have found that AMPK increases levels of GLUT4 to stimulate glucose uptake by muscle cells [17].

Skeletal muscle, playing an important role in metabolism, is responsible for energy expenditure in the body and is an important site for glucose transport and fatty acid oxidation [18]. Most of the glucose is stimulated by insulin to be metabolized in skeletal muscle tissue, ant at the same time, skeletal muscle participates in heat production and is an important organ for the transport of triglycerides [19, 20]. Glycolipid metabolism in skeletal muscle plays an important role in the pathogenesis of peripheral IR in diabetes [21]. Reasons such as overeating or lack of exercise can lead to excessive accumulation of lipids in skeletal muscle, causing skeletal muscle oxidative dysfunction and decreased metabolic capacity. Loss of muscle mass or dysfunction can also induce diabetes [22]. Therefore, the regulation of skeletal muscle metabolism has become an important research direction for the prevention and treatment of diabetes [23, 24].

Currently, the treatment for prediabetic patients is mainly through lifestyle intervention and hypoglycemic drugs. Although lifestyle intervention is simple and economical, it is difficult to achieve results in a short period time. A follow-up study showed that after six years of lifestyle intervention, many prediabetic patients still developed diabetes [25]. In addition, although drug intervention could effectively control the disease, the adverse reactions caused by metformin and other drugs, such as hypoglycemia, have increased the burden on patients. There is still no effective drug for the treatment of prediabetes. However, traditional Chinese medicine. Huidouba (HDB), also known as Bikoudai, is a nest built by *Atypus karschi Doenitz* at the roots of old tea trees. It has been passed down from generation to generation in the Mount Emei area of Sichuan for the treatment of diabetes and its complications [26]. In recent years, many studies have shown that the polysaccharides in HDB could inhibit the activity of α-glucosidase, ease the digestion and absorption of food by the stomach, reduce food intake, and also remove oxygen-free radicals, alleviate oxidative stress damage, and improve the body’s metabolism of glucose and lipids [27, 28]. Our previous research on this topic showed that, HDB could significantly reduce blood glucose in *db/db* mice, repair impaired glucose tolerance, alleviate IR, and improve diabetic nephropathy [26, 29]. Therefore, HDB has clinical research significance. Currently, the relationship between HDB and prediabetes is little known. As a good medicine for treating diabetes, we will explore whether HDB could treat prediabetes, thereby reversing the occurrence and development of T2DM, and provide an experimental basis for the clinical promotion of the drug.

In this study, by observing the effect of HDB on glucose and lipid metabolism and skeletal muscle in prediabetic C57BL/6J model mice, we explored the mechanism of HDB in treating prediabetes. Firstly, by giving a high-fat diet to replicate the prediabetic model, observe the changes in FBG in the model, and explore the regulation of glucose and lipid metabolism in the body by exploring the aspects of glucose tolerance, insulin tolerance, lipid metabolism, muscle tissue morphology, and muscle glycogen content, etc. Based on these, the western blotting method was used to analyze the expression changes of related signaling pathway proteins in skeletal muscle, revealing the molecular mechanism of the effect of HDB on prediabetes. This study provides an experimental basis for treating prediabetes by traditional Chinese medicine HDB.

## Materials

### Animals

Seventy SPF grade healthy male C57BL/6J mice (6 weeks old, with a weight of 20 ± 2 g) were purchased from SiPeiFu (Beijing) Biotechnology Co., Ltd. (No. SCXK (Beijing) 2019-0010). The breeding environment was kept at a constant temperature of 22–24 °C, relative humidity of 50–60%, and 12 h/12 h day and night alternation. This experiment was approved by the Ethics Committee of Experimental Animals of Minzu University of China (No. ECMUC2021002AO).

### Drugs and reagents

HDB is produced in Shuangfu Town, Emeishan City, Sichuan Province. It was picked in mid- and late June 2019, and it was sun-dried. It was identified as authentic by Professor Li Xin of Jilin Agricultural University.

High-fat feed was purchased from SiPeiFu Biotechnology Co., Ltd. (Beijing, China). Metformin hydrochloride was purchased from Source Leaf Biotechnology Co., Ltd (Shanghai, China). Blood glucose meters and blood glucose test strips were purchased from Sannuo (Hunan, China). Total cholesterol (TC, A111-1-4), total triglyceride (TG, A110-1-1), low-density lipoprotein (LDL-C, A113-1-1), high-density lipoprotein (HDL-C, A112-1-1), free fatty acid (FFA, A042-2-1), and lactate dehydrogenase (LDH, A020-2-2) detection kits were purchased from Nanjing Jiancheng Bioengineering Institute (Nanjing, China). Pierce BCA protein assay kit was purchased from Thermo Scientific (WL338065, Rockford, U.S.A). Antibodies against AMPK, p-AMPK, PPARα, PGC-1α, GLUT-4, β-actin and horseradish peroxidase (HRP)-conjugated goat anti-rabbit secondary antibodies were purchased from ABclonal Biotechnology Limited Company (Wuhan, China).

## Methods

### Drug preparation

For the preparation of the water extract of HDB, the preparation process parameters of HDB were summarized according to the local medication. We took 1 kg of HDB, added water 10 times the amount of the medicinal material, soaked the medicinal material for 2 h, and extracted it by boiling and refluxing (100 °C) three times, 2 h each time. The filter residue was filtered, ethanol was added to the extract until the concentration of ethanol reached 90%, alcohol precipitation was done at 4 °C for 12 h, and concentration was done under reduced pressure at 70 °C. The residue was then concentrated and dried under vacuum and reduced pressure to obtain powder of HDB by water extraction and alcohol precipitation.

### Establishment of model and treatment with drugs

After 1 week of adaptive feeding of C57BL/J6 mice, the mice were divided into six groups by the random number table method. The normal control group (n = 10) was given D12450J feed, while the prediabetic modeling group (n = 60) was given D12492 feed for 12 weeks. We checked FBG every 4 weeks. By the 12th week, FBG ≥ 6.1 mmol/L and blood glucose 7.8 ~ 11.0 mmol/L after load based on 2 h oral glucose tolerance test (OGTT) were regarded as successful modeling [30]. Among the 60 mice, 42 successfully established the prediabetic modeling, and the modeling success rate was 70%. There were seven mice’s blood glucose did not rise, and the blood glucose of 11 mice had reached the standard of diabetes (after 2 h glucose load blood glucose > 11.0mmol/L). The successfully modeled mice were divided into five groups: (1) model group (prediabetes, n = 10), distilled water; (2) metformin group (n = 8), treated with metformin hydrochloride tablets (200 mg/kg/d); (3) HDB-low group (HDB50, n = 8), given HDB 50 mg/kg/d dose; (4) HDB-intermediate group (HDB100, n = 8), given HDB 100 mg/kg/d dose; and (5) HDB-high group (HDB200, n = 8), given a dose of 200 mg/kg/d. No experimental mice died. Drugs were chronically given through intragastric administered to mice at 9:00 ~ 11:00 am for 6 weeks. During the dosing period, all prediabetic model mice remained on a high-fat diet. Mice were euthanized on the last day of the experiment, and mice blood and gastrocnemius muscle samples were collected for follow-up experiments.

### Blood glucose monitoring

We performed FBG testing every 4 weeks. OGTT and insulin tolerance test (ITT) experiments were performed on the 18th week. For the OGTT experiment, the mice were fasted for 12 h, a 50% glucose solution (2 g/kg) was administered by gavage, and the blood glucose at the tail tip was measured at 0, 30, 60, 90, and 120 min. We plotted the obtained data as a curve and then calculated the area under the curve (AUC). For the ITT experiment, the mice were not fasted, the insulin solution (0.75 U/kg) was injected intraperitoneally, and the blood glucose at the tail tip was measured at 0, 30, 60, 90, and 120 min. We plotted the obtained data as a curve and then calculated the area under the curve (AUC).

### Detection of TG, LDL-C, HDL-C, FFA, and LDH in serum

The mice serum was centrifuged at 3500 r/min for 15 min at 4 °C to separate serum samples. The contents of TG, HDL-C, LDL-C, FFA, and LDH in the serum of mice were detected according to the assay methods specified in the instructions of the kit respectively.

### Detection of TC and TG in muscle tissues

Muscle tissue was processed according to the manufacturer’s instructions on the kit. The corresponding protein concentrations were determined using the BCA kit. Then, according to the kit instructions, the content of TC and TG in mice muscle tissue was determined.

### HE staining

The gastrocnemius muscle tissue of mice was immersed in a 4% paraformaldehyde fixative solution for 2 days; cut out into 0.7 cm × 0.7 cm squares, embedded in paraffin, sliced, dewaxed, and transparentized; and hematoxylin and eosin (HE)-stained. The slides were immersed in graded ethanol (75%, 85%, 95%, 100%) for dehydration, transparent to xylene, and then two to three drops of neutral gum were added to the middle of the slides. We covered the slides with a coverslip to avoid the generation of air bubbles, and the pathological changes in gastrocnemius muscle were observed under a light microscope.

### Oil red O staining

After taking out the frozen sections of gastrocnemius muscle tissue, we placed them at room temperature for 30 min to allow the sections to return to room temperature. The slices were washed twice in distilled water for 5 min each time. The sections were placed in 60% isopropanol for 2 min of color separation. After the color separation had been completed, the sections were dried at room temperature, placed in filtered Oil red O staining solution, and stained for 5 min. We tinted the sections with 60% isopropanol and observed the color of the lipid droplets under a microscope. After toning, we immediately put them in distilled water to wash off the isopropyl alcohol and washed them twice for 5 min each time. We loaded the water-soluble mounting medium on one side of the slide with a plastic-tip dropper and covered it with the coverslip slowly, avoiding air bubbles. Changes in lipid droplets in gastrocnemius muscle tissue could be observed by imaging under a light microscope overnight.

### PAS staining

The fixed muscle tissue was dehydrated with low-concentration ethanol, embedded in xylene transparent paraffin, sliced, and dried; then the tissue was hydrated with gradient concentration ethanol, soaked in periodic acid solution for 30 min, immersed in Schiff solution, and protected from light at room temperature. Finally, we waited for glycogen to appear. After coloring, washing, dehydration, and washing, the sections were sealed with neutral glue, and the distribution of muscle glycogen was observed under an optical microscope.

### Western blotting assay

The lysate was added to the gastrocnemius muscle tissue of mice and then lysed on ice for 20 min. Then we centrifuged at 4 °C and 12,000 rpm/min for 15 min and used the supernatant for subsequent experiments. Fifty micrograms of protein samples were taken for sodium dodecyl sulfate-polyacrylamide gel electrophoresis (SDS-PAGE) and then transferred to a polyvinylidenefluoride (PVDF) membrane with 5% skim milk powder, which was blocked with TBST at room temperature for 2 h. Primary antibodies (AMPK, p-AMPK, PPARα, PGC-1α, GLUT-4, and β-actin, all diluted at 1:1000) were added and incubated at 4 °C overnight. After incubation, we rinsed the samples with TBST, then added HRP-labeled goat anti-rabbit IgG diluted 1:2000 as a secondary antibody, shook the samples at room temperature for 1 h, removed the PVDF membrane, rinsed the samples with TBST, exposed and developed them, and scanned and analyzed the target band.

### Statistical analysis

In this experiment, statistical product and service solutions (SPSS) 25.0 statistical software was used to analyze the data, and the measurement data were expressed as mean ± standard deviation (Mean ± SD). One-way ANOVA was used for the comparison of means among multiple groups. When the variance was homogeneous, the LSD and SNK methods were used for pairwise comparison among multiple groups. When the variance was unequal, the Dunnett T3 method was used. *P* < 0.05 was considered to be a significant difference. GraphPad Prism 8.0.1 software (San Diego, CA, USA) was used to draw figures.

## Results

### Changes in general characteristics in model mice

**Fig. 1:**
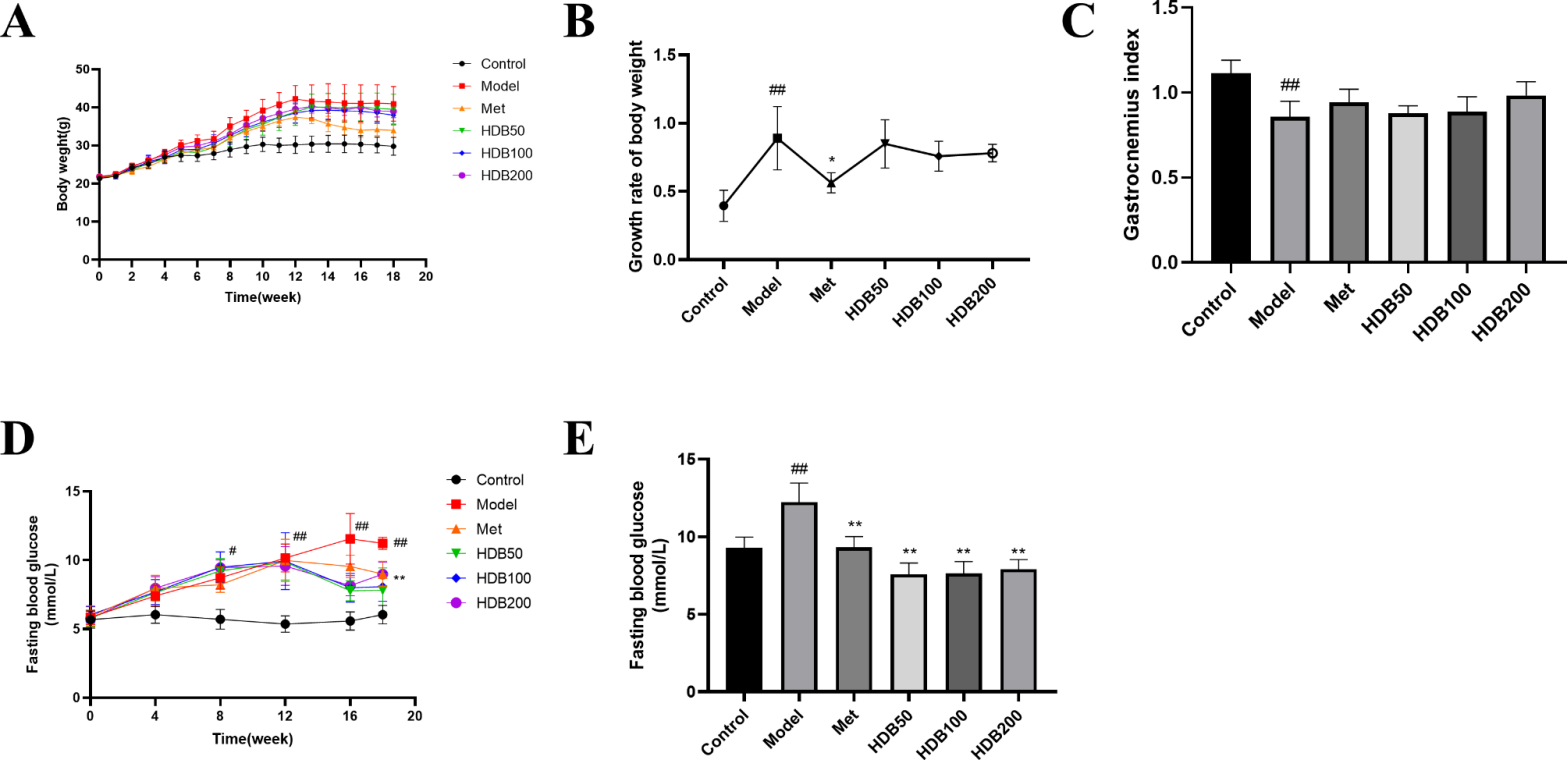
Effects of HDB treatment on body weight, fasting blood glucose, and gastrocnemius muscle index in prediabetic model mice. (**A**) Weight dynamic curve. (**B**) Growth rate of body weight. (**C**) Gastrocnemius index. (**D**) Dynamic curve of fasting blood glucose. (**E**) Fasting blood glucose 4 weeks after dosing. Data are presented as mean ± SD, n = 8. ^#^*P* < 0.05, ^##^*P* < 0.01 vs. control group; ^*^P < 0.05, ^**^P < 0.01 vs. model group

After the model had been established, the weight of the mice in the normal group was relatively stable, and the weight of the mice in the model group and each administration group continued to increase (Fig. 1A). Metformin had an inhibitory effect on the weight gain of mice, but there was no significant difference between the administration groups and the model group (Fig. 1B). In addition, the gastrocnemius muscle index of the model mice was significantly decreased, and there was an increasing trend after high-dose HDB treatment, but there was no significant difference (Fig. 1C). Figure 1D shows that the FBG of the model mice gradually increased during the modeling period. After 4 weeks of administration, the FBG of the mice in the model group was significantly increased (*P* < 0.01), which was reversed by metformin and HDB treatment, and the FBG was significantly decreased (*P* < 0.01). HDB treatment could significantly suppress blood sugar elevation (Fig. 1E).

### Effect of HDB on glucose tolerance and insulin tolerance

**Fig. 2:**
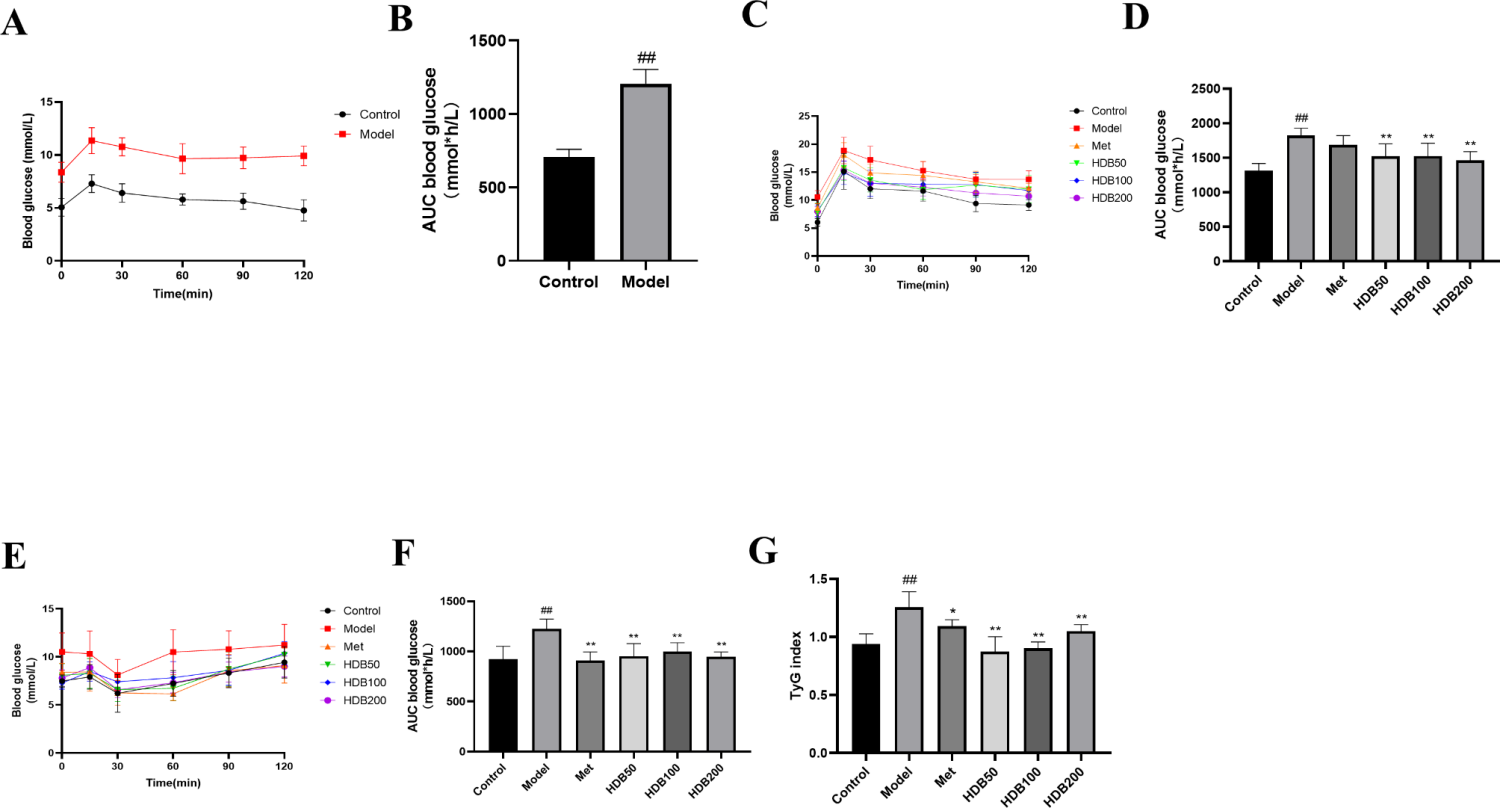
HDB enhanced glucose tolerance in model mice. (**A**) OGTT (12th week). (**B**) Area under the OGTT curve. (Control: n = 10, Model: n = 42) (**C**) OGTT (18th week). (**D**) Area under the OGTT curve. (**E**) ITT (18th week). (**F**) Area under the ITT curve. (**G**) TyG index. Data are presented as mean ± SD, n = 8. ^#^*P* < 0.05, ^##^*P* < 0.01 vs. control group; ^*^P < 0.05, ^**^P < 0.01 vs. model group

The OGTT and the area under the OGTT curve (AUC) showed that after 12 weeks of modeling, the model mice we screened had apparent impaired glucose tolerance (*P* < 0.01) (Fig. 2A, B). After six weeks administration, each HDB administration group could significantly improve the impaired glucose tolerance of model mice (*P* < 0.01) (Fig. 2C, D). At the same time, the ITT and the ITT’s AUC showed that the insulin tolerance of the prediabetic model mice was significantly reduced (*P* < 0.01). However, metformin and HDB could effectively improve the impaired insulin tolerance of model mice (*P* < 0.01) (Fig. 2E, F). Moreover, by detecting serum triglycerides and calculating the corresponding triglyceride glucose index (TyG = ln ((TG*FBG)/2), we found that the TyG of the prediabetic model mice was significantly increased (*P* < 0.01), and there was significant IR. After HDB treatment, IR improved (*P* < 0.01) (Fig. 2G). These data indicate that HDB can improve glucose tolerance and IR in prediabetic model mice.

### Effect of HDB on lipid metabolism in serum

**Fig. 3:**
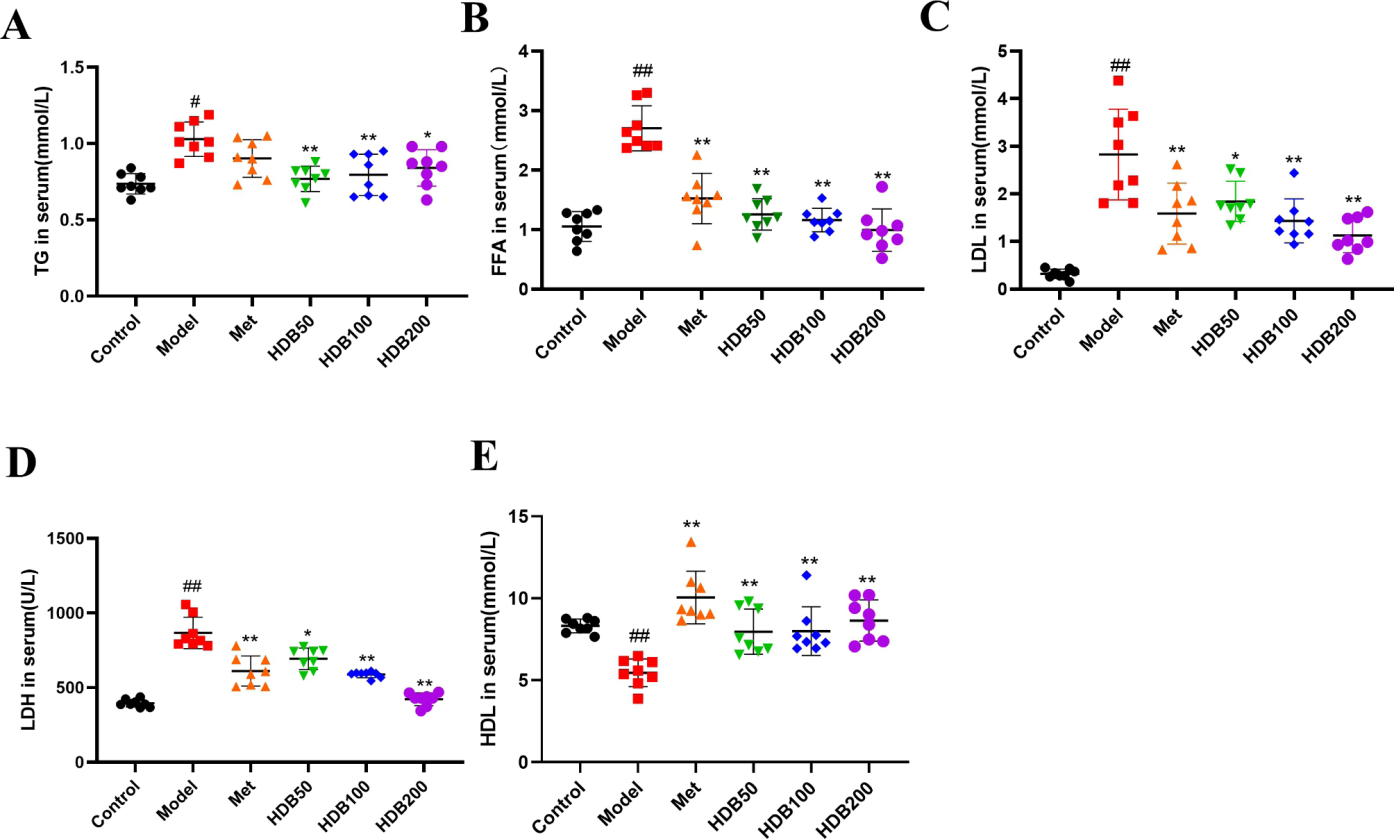
HDB alleviated dyslipidemia in prediabetic mice. The levels of (**A**) TG, (**B**) FFA, (**C**) LDL, (**D**) LDH, and (**E**) HDL. Data are presented as mean ± SD, n = 8. ^#^*P* < 0.05, ^##^*P* < 0.01 vs. control group; ^*^P < 0.05, ^**^P < 0.01 vs. model group

As shown in Fig. 3, serum TG, FFA, LDL, and LDH levels in mice were significantly increased (*P* < 0.01) (Fig. 3A-E), while HDL content was decreased (*P* < 0.01) (Fig. 3F). After HDB treatment, the contents of TG, FFA, LDL and LDH were significantly decreased (*P* < 0.05), and the content of HDL was significantly increased (*P* < 0.01). These results indicate that HDB can effectively reverse dyslipidemia in prediabetic mice.

### Effects of HDB on muscle histology and lipid accumulation in muscle tissue

**Fig. 4:**
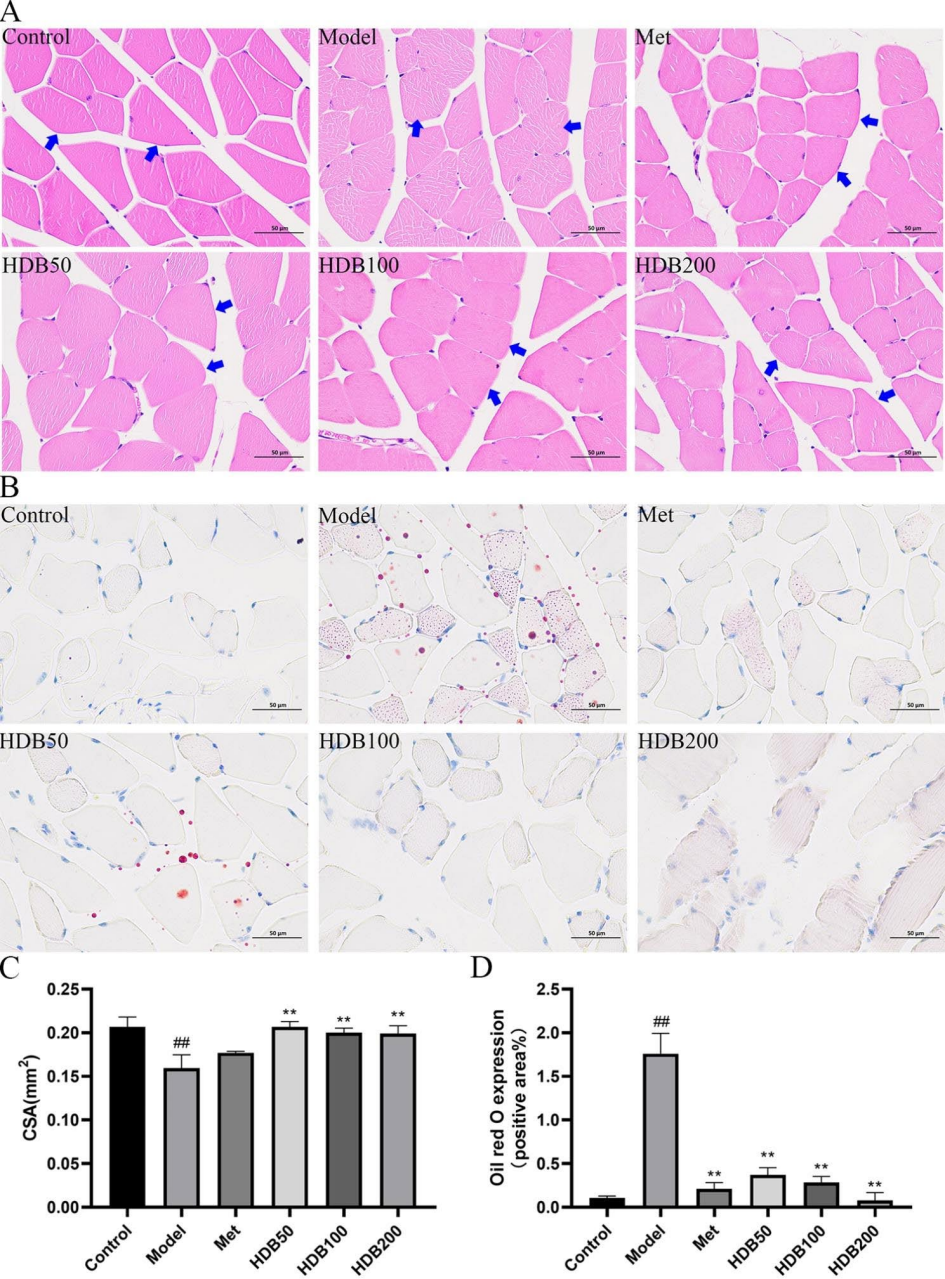
HDB can improve muscle tissue damage and attenuate muscle lipid deposition in prediabetic model mice. (**A**) HE staining (400×). The skeletal muscle tissue structure of the mice in the control group was normal, the sarcolemma was intact, no obvious lesions were found, and the cell nuclei were arranged neatly under the sarcolemma. The skeletal muscle structure of the model mice was abnormal, the number of nuclei increased and was pathologically aggregated, the arrangement of muscle cells was disordered, the space between the sarcolemma increased. The histopathological structure of skeletal muscle in the HDB and metformin administration groups was improved, muscle fibers were gradually complete and regular, muscle cells were gradually arranged, skeletal muscle tissue structure was basically restored. (**B**) Oil red O staining (400×). (**C**) The CSA of muscle fibers. (**D**) Oil red O expression. Data are presented as mean ± SD, n = 3. ^#^*P* < 0.05, ^##^*P* < 0.01 vs. control group; ^*^P < 0.05, ^**^P < 0.01 vs. model group

**Fig. 5:**
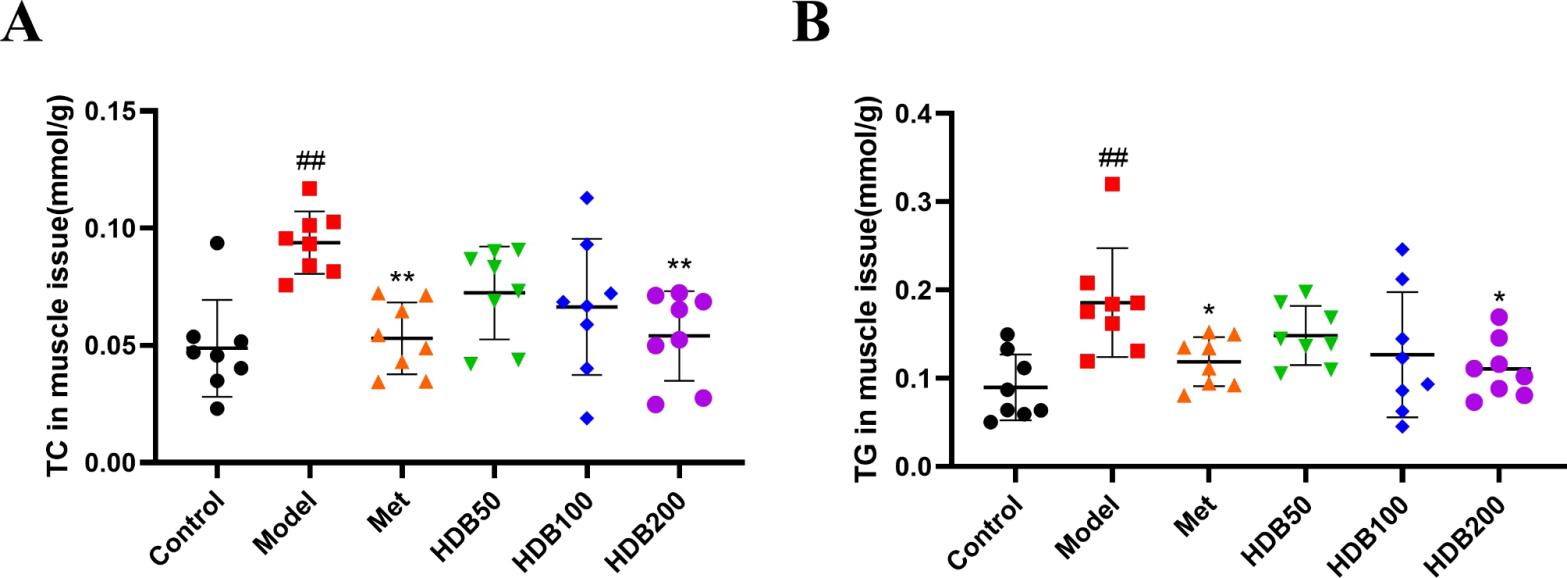
HDB alleviates muscle lipid deposition in prediabetic model mice. (**A**) The level of TC in muscle tissues. (**B**) The level of TG in muscle tissues. Data are presented as mean ± SD, n = 8. ^#^*P* < 0.05, ^##^*P* < 0.01 vs. control group; ^*^P < 0.05, ^**^P < 0.01 vs. model group

As shown in Fig. 4A and C, the histopathological results of the skeletal muscle tissue showed that the skeletal muscle tissue structure of the mice in the control group was normal, the sarcolemma was intact, no obvious lesions were found, and the cell nuclei were arranged neatly under the sarcolemma. The skeletal muscle structure of the model mice was abnormal, the number of nuclei increased and was pathologically aggregated, the arrangement of muscle cells was disordered, the space between the sarcolemma increased, and the cross-sectional area (CSA) of muscle fibers decreased significantly (*P* < 0.05). After 6 weeks of administration, the histopathological structure of skeletal muscle in the HDB and metformin administration groups was improved, muscle fibers were gradually complete and regular, muscle cells were gradually arranged, skeletal muscle tissue structure was basically restored, and the CSA of muscle fibers increased significantly (*P* < 0.01). Oil red O staining (Fig. 4B and D) showed no lipid accumulation in control mice muscle tissue. Interestingly, there was diffuse fat accumulation in the muscle tissue of the model mice. After HDB intervention, the structure of muscle tissue was improved, and the degree of lipid bullae and lipid aggregation was reduced (*P* < 0.05). More importantly, we found that the contents of TC and TG in the muscle tissue of model mice (Fig. 5A, B) were significantly increased (*P* < 0.01). Metformin and HDB200 significantly decreased liver TC and TG levels (*P* < 0.01, *P* < 0.05). This indicates that HDB can reduce fatty deposits in muscle tissue and improve muscle tissue structure.

### Effects of HDB on glycogen in muscle tissue

**Fig. 6:**
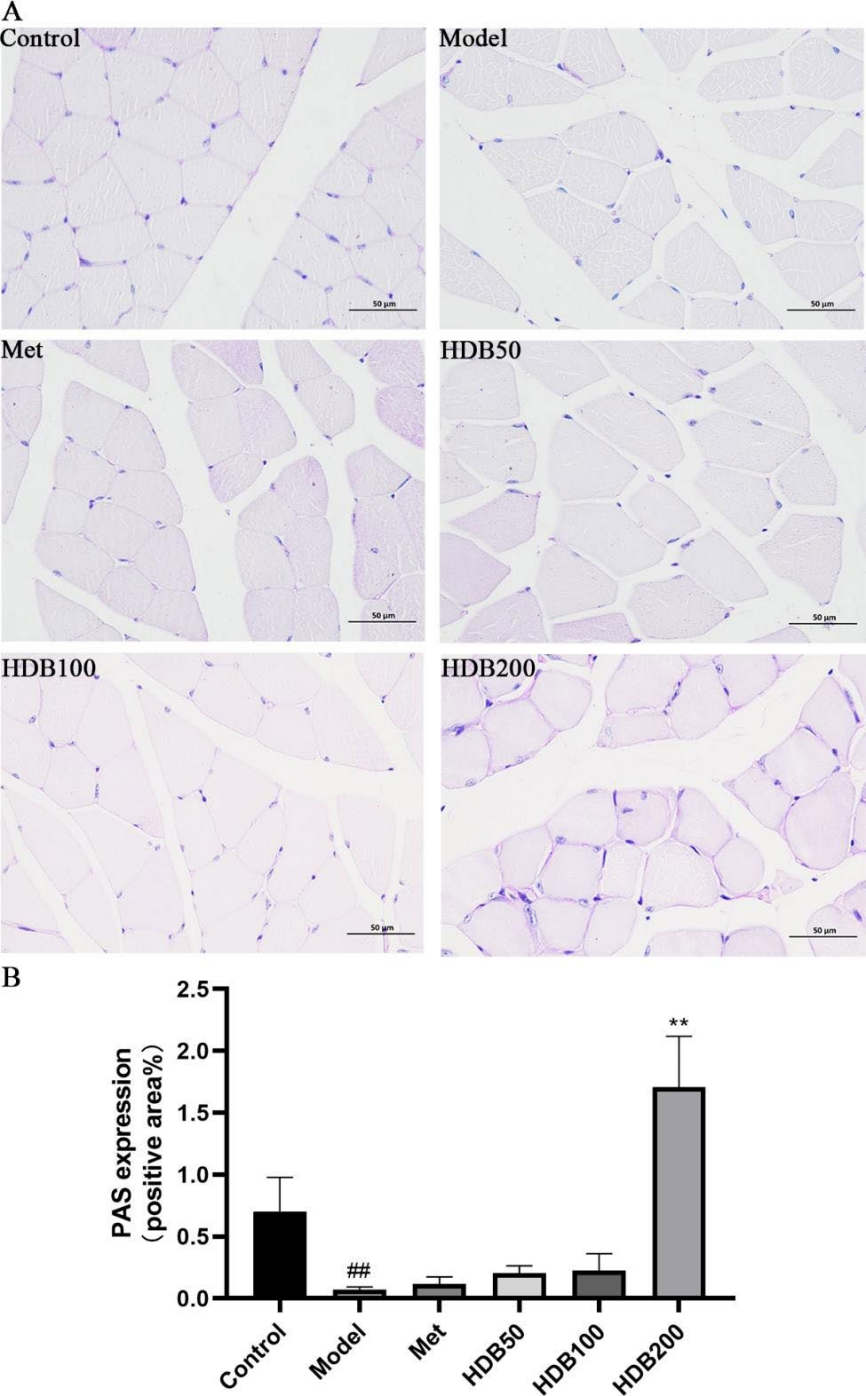
HDB promotes muscle glycogen accumulation in prediabetic model mice. (**A**) PAS staining (400×). (**B**) PAS expression. Data are presented as mean ± SD, n = 3. ^#^*P* < 0.05, ^##^*P* < 0.01 vs. control group; ^*^P < 0.05, ^**^P < 0.01 vs. model group

As shown in Fig. 6A, B, compared with the control group, the expression rate of PAS staining-positive cells in the prediabetic model mice was significantly decreased (*P* < 0.01), while the expression rates of positive cells in the HDB200 administration groups were significantly increased (*P* < 0.01). This indicates that the HDB of 200 mg/kg can promote the accumulation of glycogen in muscle cells and regulate blood sugar disturbances.

### Effects of HDB on the expression of glucose metabolism-related proteins

**Fig. 7:**
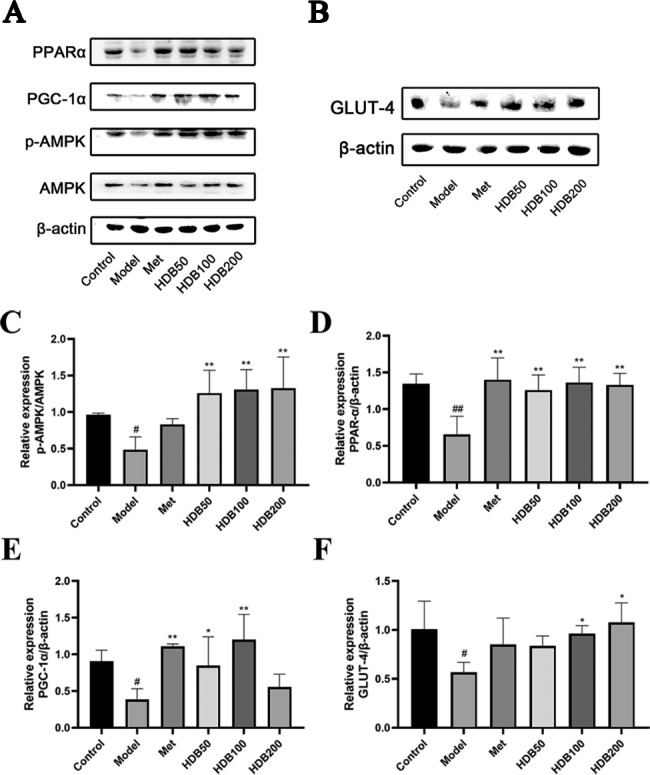
HDB increased proteins expression in the muscle of model mice. (**A-F**) The expression of AMPK, p-AMPK, PGC-1α, PPARα, and GLUT-4 were examined by western blot assay. Data are presented as mean ± SD, n = 3. ^#^*P* < 0.05, ^##^*P* < 0.01 vs. control group; ^*^P < 0.05, ^**^P < 0.01 vs. model group

To verify whether HDB regulates glucose metabolism through the AMPK/PGC-1α/PPARα pathway as well as GLUT-4, we tested key proteins in this pathway, such as p-AMPK, AMPK, PGC-1α, PPARα, and GLUT-4. Figure 7 shows that the expression of p-AMPK/AMPK, PGC-1α, PPARα, and GLUT-4 in the muscle of model mice was significantly decreased (*P* < 0.01), while HDB intervention upregulated the expression of these proteins (*P* < 0.01). These results indicate that HDB could promote glucose absorption and regulate lipid biosynthesis by activating the AMPK/PGC-1α/PPARα pathway and upregulating the expression of GLUT-4, thereby improving the disturbance of glucose and lipid metabolism in the muscles of prediabetic mice.

## Discussion

Prediabetes is a key node in the prevention of T2DM, and IGT and IFG are typical manifestations of prediabetes [3]. The metabolism of skeletal muscle is the key pathogenesis of T2DM development [23]. Studies have found that metabolic disturbances in skeletal muscle in patients with prediabetes can aggravate IR, and mitochondria in skeletal muscle are damaged, which in turn promote the development of T2DM [31]. Therefore, prediabetes and skeletal muscle metabolism are important components in the prevention of T2DM progression.

In this study, prediabetic model mice were successfully established by feeding C57BL/J6 mice with a high-fat diet for 12 weeks. The model group showed high FBG levels. Adam et al. found that hyperglycemia was a driver of prediabetes progression to T2DM [30]. Our data show that after 6 weeks of HDB intervention, FBG was significantly reduced in prediabetic model mice, while IR and glucose tolerance were significantly improved. HDB showed the same ability to regulate glucose metabolism as metformin, and each HDB administration group showed a good hypoglycemic effect. Zhang et al. found that polysaccharide-containing drugs could significantly improve blood sugar disturbances [32]. These results indicate that the effects of HDB on glucose tolerance and insulin tolerance in prediabetic model mice may be related to its polysaccharide composition.

Many studies have shown that prediabetes is also accompanied by lipid disorders, the TG/HDL ratio and LDL/HDL ratio are significantly increased, and HDL is significantly decreased [33]. This change is inextricably linked to IR. High TG triggers lipotoxicity and directly promotes inflammation and endoplasmic reticulum stress, leading to IR [34]. Likewise, IR increases TG synthesis, resulting in hypertriglyceridemia, and a low HDL concentration [35, 36]. A major factor in this mechanism is the accelerated rate of lipolysis of TG-derived FFA stored in adipose tissue, resulting in increased FFA flux to the liver [37]. This is consistent with the observation of increased serum FFA concentrations in model mice in this experiment. Furthermore, increases in plasma FFA correlate with IR [38, 39] through intramyocyte and intrahepatic accumulation of TG and other metabolites [40]. Our data indicate that metformin has a good effect in regulating blood lipids. Low, medium, and high doses of HDB also have a good effect in alleviating some abnormal blood lipid metabolism indices.

Numerous studies have found that skeletal muscle is indispensable for the development of IR [41]. In the IR state, insulin-stimulated glucose disposal in skeletal muscle is significantly impaired, including impaired glucose transport and glucose phosphorylation, as well as reduced glucose oxidation and glycogen synthesis [42–45]. This is consistent with the significant reduction in muscle glycogen in model mice observed in our experiments. The occurrence of skeletal muscle IR may be related to intracellular fat content and fatty acid metabolites [46, 47]. If the rate of fat supply exceeds the demand for fat oxidation, the muscle redirects the fat entering the cell for TG synthesis, resulting in a significant increase in TG content in skeletal muscle [48]. In addition, skeletal muscle with IR decreases insulin sensitivity and increases plasma FFA concentrations, while increased FFA inhibits insulin-stimulated glucose processing in skeletal muscle, reduces sugar uptake, and reduces glycogen synthesis [49–51]. More interestingly, the study found that overexpression of the PGC-1α gene in mouse skeletal muscle increased the expression of various proteins involved in fat oxidation and glucose transport, and increased insulin-stimulated glucose uptake by approximately 35% [52]. Our data indicate that the application of HDB can effectively reduce TC and TG levels in muscle tissue and promote glycogen accumulation. The HDB of 200 mg/kg was comparable to the metformin. This indicates that HDB can regulate lipid metabolism in skeletal muscle of prediabetic model mice and promote gluconeogenesis. This may be related to IR.

Metabolic disturbances in skeletal muscle cannot be ignored in prediabetes. As early as the 1990s, AMPK was recognized as a key regulator of skeletal muscle metabolism, which promotes ATP-generating catabolism associated with glucose and fat oxidation [53–55]. The inhibition of AMPK reduces glucose uptake by skeletal muscle and reduces muscle glycogen [56]. Therefore, the activation of AMPK is critical for the metabolism of skeletal muscle. Studies have found that the activation of the AMPK/PGC-1α/TFAM signaling pathway can enhance mitochondrial biosynthesis in skeletal muscle, increasing glucose uptake and ATP production [57]. PPARα is another important regulator of glucose and lipid metabolism, which can be activated by PGC-1α. More interestingly, PPARα is also involved in the activation of AMPK, as the phosphorylation of AMPK can upregulate PPARα [11, 15]. AMPK and PGC-1α gene expression was significantly higher in HDB-treated mice compared with prediabetic model mice. Significant increases in phosphorylation and total AMPK protein were observed in the HDB-treated group. Thus, the expression of PPARα can be regulated owing to AMPK phosphorylation and the activation of PGC-1α by HDB. Based on this, HDB enhanced AMPK phosphorylation-mediated PPARα expression in skeletal muscle, which was reflected as increased AMPK-PGC-1α-PPARα gene expression in skeletal muscle. Our observations are similar to those reported for the AMPK/PGC-1α/TFAM pathway. This mechanism of HDB shares some similarities with the effect of metformin against T2DM, where metformin is an AMPK activator that increases PGC-1α expression [58]. In addition, the ability of AMPK to promote glucose uptake in skeletal muscle can also occur acutely through the transfer of GLUT4 from intracellular storage vesicles to the plasma membrane, and can also occur chronically through upregulation of GLUT4 expression [59]. This is consistent with the upregulation of GLUT-4 expression we observed. Therefore, the activation of the AMPK/PGC-1α/PPARα pathway to regulate skeletal muscle glucose and lipid metabolism disorders is an important measure for the treatment of prediabetes. The above results indicate that HDB can regulate and improve glucose and lipid metabolism through the activation of the AMPK/PGC-1α/PPARα pathway and the upregulation of GLUT-4 expression, and ultimately improving the symptoms of prediabetic model mice. Based on this, we believe that the relieving effect of HDB on prediabetes may be related to the AMPK/PGC-1α/PPARα signaling pathway and GLUT-4 protein.

This study still had some limitations, such as some inhibitors or activators didn’t be added to inhibit or activate the expression of one of these proteins. In the follow-up research, it will be necessary to use inhibitors and agonists to further explore the role of the AMPK/PGC-1α/PPARα pathway in the improvement of skeletal muscle metabolism by HDB, and continue to explore the target of HDB. In addition, further experimental validation is required to identify the core mechanisms for the prevention and treatment of prediabetes. The effective substances that have a therapeutic effect on HDB also need to be verified by follow-up experiments.

## Conclusions

In this study, we focused on skeletal muscle to elucidate the effect of HDB in prediabetic model mice. By regulating FBG, lipid metabolism, as well as lipid aggregation and glycogen accumulation of skeletal muscle, HDB improved the body’s IR and skeletal muscle metabolism disorder, possibly by activating the AMPK/PGC-1α/PPARα signaling pathway and up-regulating GLUT-4 protein in skeletal muscle. This suggests that the traditional Chinese medicine HDB is a promising drug to treat prediabetes and prevent the disease from developing into T2DM. Our results provided an experimental basis for treating prediabetes by traditional Chinese medicine HDB.

## Electronic supplementary material

Below is the link to the electronic supplementary material.

Supplementary Material 1

## Data Availability

The datasets used and/or analyzed during the current study are available from the corresponding author on reasonable request.

## Abbreviations

AMPK: Adenosine 5’-monophosphate-activated protein kinase
FBG: Fasting blood glucose
FBG: Fasting plasma glucose
FFA: Free fatty acid
GLU: Glucose
GLUT4: Glucose transporter-4
HDB: Huidouba
HDL: High-density lipoprotein
IFG: Impaired fasting glucose
IGR: Impaired glucose regulation
IGT: Impaired glucose tolerance
INS: Insulin
IR: Insulin resistance
ITT: Insulin tolerance test
LDH: Lactate dehydrogenase
LDL: Low-density lipoprotein
OGTT: Oral glucose tolerance test
PGC-1α: Peroxisome proliferator-activated receptor γ coactivator-lα
PPARα: Peroxisome proliferator-activated receptor-α
T2DM: Type 2 diabetes mellitus
TC: Total cholesterol
TG: Triglycerides
