# Supplementary figures and images for: The role of Huidouba in regulating skeletal muscle metabolic disorders in prediabetic mice through AMPK/PGC-1α/PPARα pathway
Source: Diabetol Metab Syndr. 2023 Jul 1;15:145. doi: 10.1186/s13098-023-01097-8 (PMC10314379; doi:10.1186/s13098-023-01097-8)

Supplementary Figure. WB-original fig


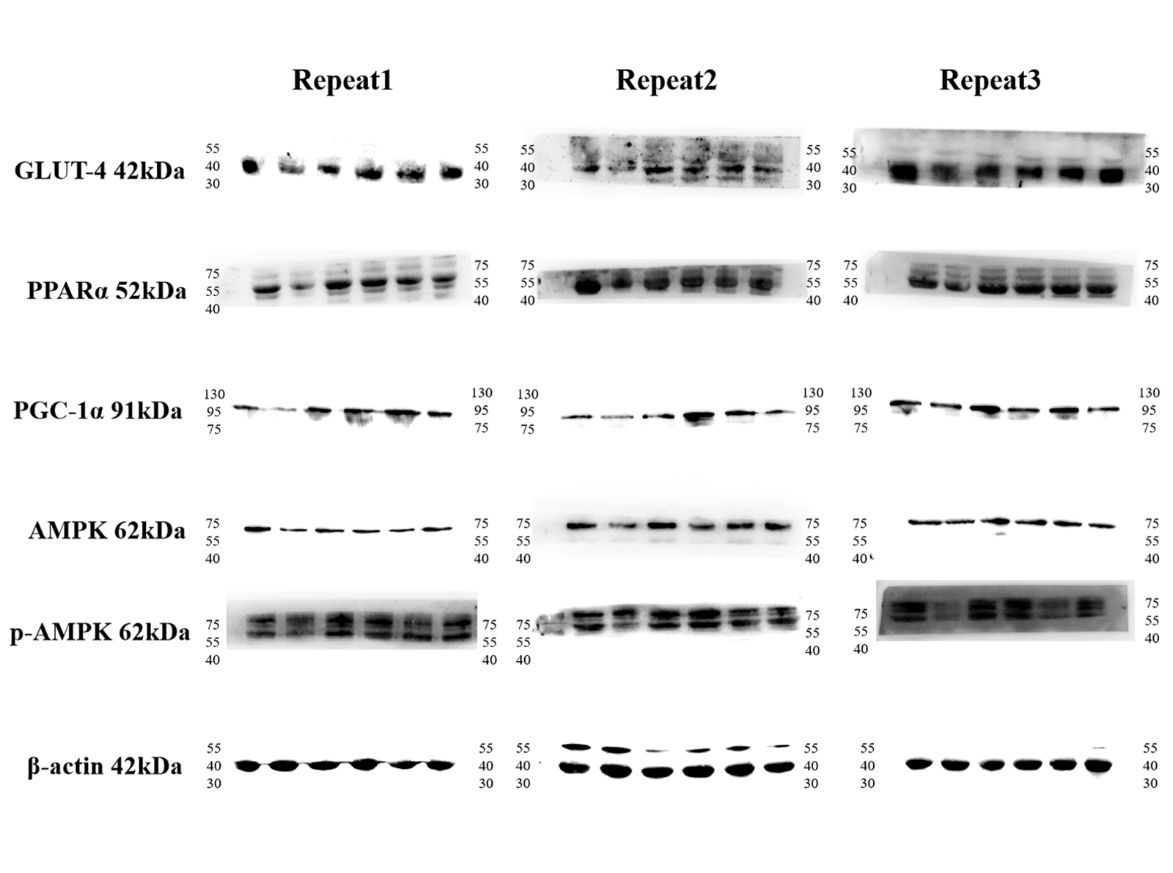

Supplement: Supplementary file 1 — Supplementary Material 1 [file 13098_2023_1097_MOESM1_ESM.docx]
